# Unusual rotavirus genotypes among children with acute diarrhea in Saudi Arabia

**DOI:** 10.1186/s12879-015-0923-y

**Published:** 2015-04-17

**Authors:** Mahmoud Aly, Aisha Al Khairy, Sameera Al Johani, Hanan Balkhy

**Affiliations:** King Abdullah International Medical Research Centre, P.O. Box 22490, Riyadh, 11426 Kingdom of Saudi Arabia; King Saud bin Abdulaziz University for Health Sciences, Riyadh, Saudi Arabia; Department of Microbiology, King Abdulaziz Medical City, Riyadh, Saudi Arabia; Department of Infection Prevention and Control, King Abdulaziz Medical City, Riyadh, Saudi Arabia

**Keywords:** Rotavirus, Gastroenteritis, Diarrhea, Epidemiology, VP4, VP7, KSA

## Abstract

**Background:**

Human rotavirus A (human RV-A) is the most common cause of viral gastroenteritis in infants. The objective of the study was to characterize the G and P genotypes among clinical rotavirus isolates from children with acute diarrhea admitted to a tertiary care hospital in Riyadh, Saudi Arabia.

**Methods:**

From 2011 to 2012, 541 pediatric patients with acute diarrhea were tested for rotavirus infection. RNA extractions from the fecal specimens were done by commercial kit. RT-PCR and sequencing techniques were used to detect the prevalent genotypes. Phylogenetic analysis by Maximum Likelihood method was used to study the clustering of the circulating genotypes.

**Results:**

The data showed that 171/541 (31.6%) faecal samples were positive for human RVA and majority were children aged below 2 years. From the G and P [types] detected it was seen that (a) 171 minus 43 ie. 128 rotavirus positives were G typed successfully (b) 171 minus 20 ie. 151 rotavirus positives were P typed successfully; (c) overall G [P] nature was determined for 113 rotavirus positives out of 171. VP4 genotyping showed that majority of the positives 146/151 (96.7%) were P [8]; 4/151 (2.6%) were P [4]; 1/151 (0.66%) was P [6]. The dominant strains included G1P [8] 70/113 (61.9%); G9P [8] 19/113 (16.8%); G12P [8] 7/113 (6.2%) and G3P [8] 5/113 (4.4%) while the uncommon strains detected from Saudi Arabia during the study were G1P [4] 1/113 (0.88%) and G12P [6] 1/113 (0.88%). Phylogenetic tree, based on VP4/VP7 sequence analysis, revealed that G1P [8] was distinctly related to homologous strains included in human RV-A vaccine strains. Nevertheless, the uncommon genotypes G1P [4] and G12P [6] were clustered with isolates from other countries such as Bangladesh, China, Japan, Thailand and Philippines.

**Conclusions:**

More studies will be required to further focus on newly emerging genotypes in our region together with the seasonality of rotavirus infection in the region, especially after January 2013 when the rotavirus vaccination has become part of routine childhood immunizations.

## Background

Human rotavirus A (human RV-A) is the most common pathogen causing viral gastroenteritis (VGE) [1]. Human RV-A infections are responsible for 500,000 associated deaths annually among the children worldwide. In addition, human RV-A infection causes 25-35% of all gastroenteritis hospitalized cases in children [2]. In previous work, we showed that host susceptibility to viral infections is due to genetic and environmental factors [3,4]. Infants and children are more vulnerable to human RV-A infection. More than 95% of all children under the age of 5 years were found to be infected with RV at least once; half of those were 2 years old or younger [5,6]. The incidence of human RV-A disease in industrial and developing countries is more or less the same [1,7]; however, high mortality rates mainly occur in the developing countries, particularly those with low revenues and poor health care systems [8].

Rotaviruses (RV) belong to *Reoviridae* family and are double-stranded (ds) non-enveloped RNA viruses. Their genome consists of 11 ds-RNA segments and they are classified based on 2 of these genome segments called VP4 and VP7, which encode for outer capsid proteins [9]. Group A is the most pathogenic among common human VGE pathogens. RV-As are classified into at least 27 G and 37 P types that are known to be genetically different [10,11]. Most common types, such as G1P [8], G2P [4], G3P [8], and G4P [8] are causing more than 90% of human RV-A cases in North America and Europe [12]. Despite these global trends observed, there are heterogeneous regional distributions found in terms of human RV-A types.

Currently, the data on the prevalence of human RV-A cases in Saudi Arabia are lacking, while the seasonal increases are observed usually in winter and spring time. Understanding the locally-circulating genotypes will eventually assist in the development of more effective vaccine candidates and prevent the spread of this infectious disease. The main objectives of the study were (1) to determine the prevalence of rotavirus genotypes with a focus on VP4 and VP7 genotypic characterization; and (2) to perform the human RV-A molecular epidemiology study in Saudi Arabia aiming at the possible emergence of new genotypes in the region.

## Methods

### Ethical compliance

This study was approved by the IRB Committee of King Abdullah International Medical research center (KAIMRC), National Guard Health affaires, Riyadh, KSA (reference number # RC08-112). All the samples were taken as part of standard care.

### Stool samples

A total of 541 stool samples were collected from ongoing prospective study assessing the rotavirus epidemiology and genotypes. These isolates were taken from pediatric patients with acute diarrhea in the city of Riyadh between 2011 and 2012, which represents the main urban center in Saudi Arabia. Each stool sample was immediately frozen undiluted at −20°C until use.

### Epidemiological data and data analysis

Epidemiological data including age, sex, and symptoms (diarrhoea and/or vomiting and their number of episodes), feeding type, date of onset, and date of sample collection were collected using Epic. Info vr.4 and molecular genotyping data were entered into Excel worksheets. Descriptive analysis, frequencies and percentages were calculated using SPSS vr. 20 statistical software.

### ELISA screening for rotavirus

All stool samples were screened using ImmunoCard Stat Rotavirus kit® which detects the presence of rotavirus antigen in stool based on enzyme-linked immunosorbent assay (ELISA) method. Briefly, the stool specimen was diluted 1:15 in sample diluents and thoroughly mixed. The suspension was introduced (50 μl) to the sample port of the device and rotavirus detection was carried out following the manufacturer’s instructions. In addition to the internal (test) control, we included two external controls from the previously tested (known) samples.

### RNA extraction

All positive ELISA samples were confirmed by RT-PCR. In brief, RNA was extracted from approximately 1 mg of stool sample using MagNA Pure Compact RNA Isolation Kit (Roche Diagnostics GmbH, Mannheim, Germany) and following the manufacturer’s instructions. Extracted RNA was immediately stored at - 80°C until further use.

### Reverse transcription (RT) and PCR amplification reactions

RT reactions were carried out in a final volume of 60 μl using Transcriptor First Strand cDNA Synthesis Kit (Roche Diagnostics GmbH, Mannheim, Germany) on a Tetrad 2 Peltier thermal cycler from BIO-RAD. A master mix was prepared containing 5× RT buffer, 40 U/μl RNAse inhibitor, 20U/μl Transcriptor reverse transcriptase, 10 mM each Deoxynucleotide Mix, 600 μM Random Hexamer Primer, RotaA.ext. Fwd primer 5′-TTT AAA ACG AAG TCT TCR ACA TGG AKG TYC TGT A-3′ and RotaA.ext. Rev primer 5′-TAA TTG GTG ATC TAC CAA TTC CTC CAG TTT G-3′ [13]. RNA sample (10 μl) was heated at 99°C for 10 min, placed immediately on ice, and 50 μl of master mix was then added to each tube. The temperature cycling was 50°C for 50 min, 94°C for 7 min and cDNA was amplified using RotaA.ext. forward and reverse primers [13]. The temperature cycling was 95°C for 10 min, 40 cycles of (94°C for 1 min, gradient from 45°C to 65°C for 3 min, 72°C for 1 min), and final extension at 72°C for 10 min.

### Genotyping by PCR amplification and sequencing

ELISA positive rotavirus samples (n = 171) were further confirmed by RT-PCR using primers set used for rotavirus genotyping VP4 [P] and VP7 [G] genotypes. Genotyping was performed using the following oligonucleotide primer pairs (Eurofins MWG Operon) targeting the VP4 and VP7 gene regions: VP4-F 5′-TAT GCT CCA GTN AAT TGG-3′, VP4-R 5′-ATT GCA TTT CTT TCC ATA ATG-3′, VP7-F 5′-ATG TAT GGT ATT GAA TAT ACC AC-3′ and VP7-R 5′-AAC TTG CCA CCA TTT TTT CC-3′[13,14]. Thermal cycling for the VP4 region was 95°C for 10 min, 35 cycles of (94°C for 1 min, 50°C for 1 min, 72°C for 1 min), then 72°C for 10 min. Thermal cycling for the VP7 region was 95°C for 10 min, 40 cycles of (94°C for 1 min, gradient from 45°C to 65°C for 3 min, 72°C for 1 min), then 72°C for 10 min. Amplified products were analyzed on 1.5% agarose ethidium bromide-stained gels for genotyping. All PCR products of the appropriate size were purified using 3 M sodium acetate and absolute ethanol. Purified products were confirmed by sequinning using BigDye Terminator v3.1 Cycle Sequencing Kit (Applied biosystems™ Austin, TX, USA). Thermal cycling was 96°C for 1 min, 40 cycles of (96°C for 10 min, 50°C for 5 sec, 60°C for 4 min). Amplified products for VP4 (663 bp) and VP7 (880 bp) were purified a second time using XTerminator™ and SAM™ solutions (Applied Biosystems™ Foster City, CA, USA). Purified products were sequenced using 3730xl DNA Analyzer (Applied Biosystems™, Hitachi, Tokyo, Japan).

### Assignment of the genotypes and phylogenetic analysis

All isolates sequences were analyzed in comparison to VP4 and VP7 of the international rotavirus sequences available at NCBI sequence database using BALSTn-2 and RotaC 2.0 [15] automated genotyping tool for Group A rotaviruses. Molecular phylogenetic analysis was carried out using Maximum Likelihood method based on Tamura-Nei model [16]. The tree was drawn to scale with branch lengths measured in the number of substitutions per site and was bootstrapped with 1000 replicates. The analysis involved 635 nucleotide sequences. All positions containing gaps and missing data were eliminated. There were a total of 681 positions in the final dataset. Evolutionary analyses were conducted in MEGA6 [17].

## Results

### Rotavirus occurrence and demographic data

The total number of cases collected between 2011 and 2012 was 541 and one stool sample was obtained from each child, representing one case. Of all 541 cases, 171 (30.6%) were found rotavirus positive by ELISA. The demographic data of the 541 cases are shown in Table 1.

**Table 1:** **Demographic data of the children with acute diarrhea presented between 2011–2012 at KAMC-R**

		N*	**%**
**Gender**	Male	302/540	55.9%
	Female	238/540	44.1%
**Vomiting**	No	36/540	6.7%
	Yes	504/540	93.3%
**Diarrhea**	No	12/540	2.2%
	Yes	528/540	97.8%
**Rotavirus**	Negative	370/541	68.4%
	Positive	171/541	31.6%
**Feeding Type**	Bottle only	72/537	13.4%
	Breast Only	6/537	1.1%
	Bottle and breast	112/537	20.9%
	Table Feed	213/537	39.7%
	Bottle and table feed	110/537	205%
	Breast and table feed	3/537	0.6%
	Bottle, breast and table feed	21/537	3.9%

*Total number of samples (N = 541); positive rotavirus samples in bold, some demographic data were not available.

### Seasonal incidence of rotavirus gastroenteritis within the period from 2011 to 2012

**Figure 1:**
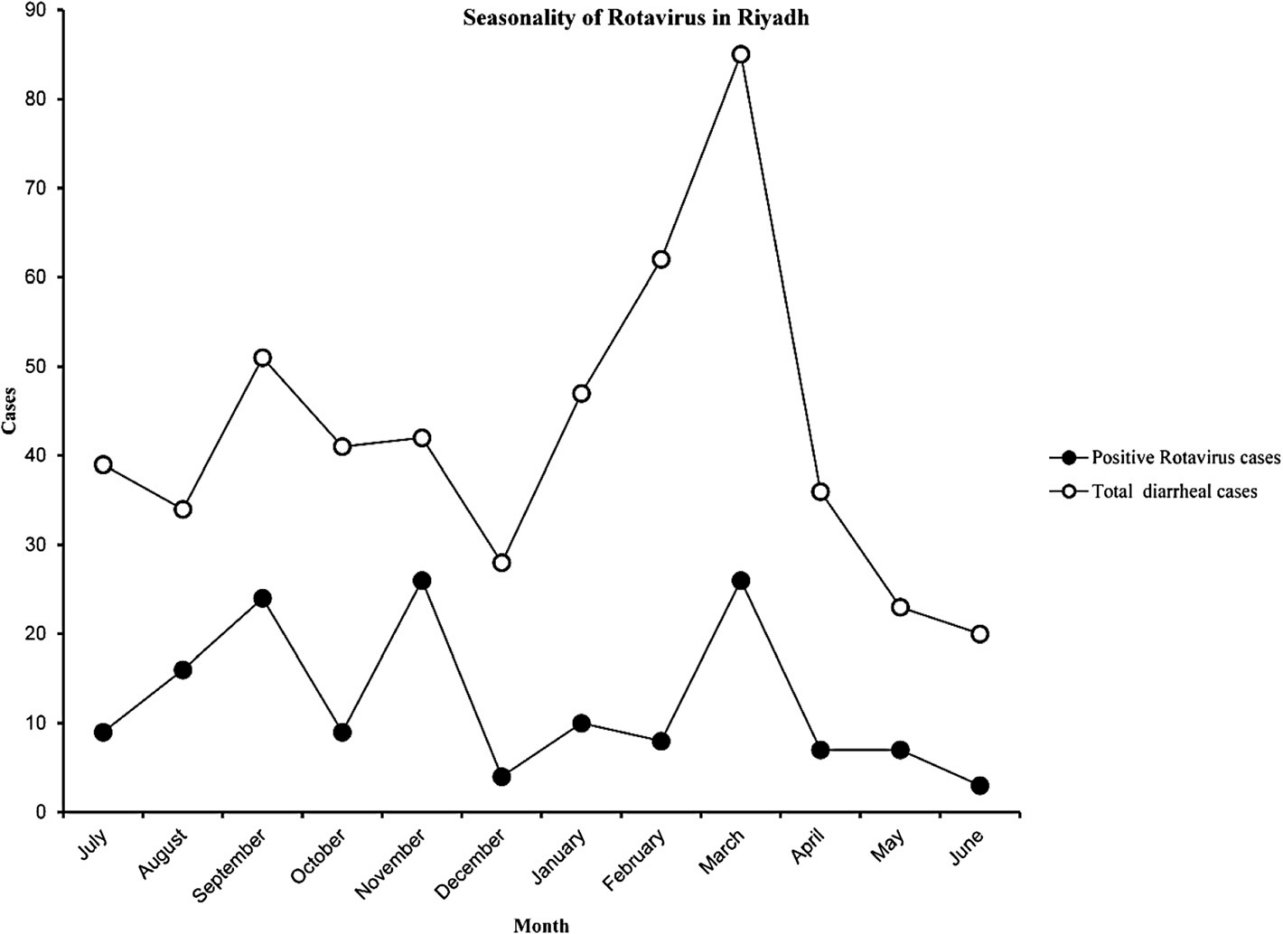
Monthly seasonality of viral diarrhea cases in Saudi children (2011–2012). [Represented by monthly positive cases variation that peaked in winter season].

The occurrence of acute gastroenteritis and the rotavirus incidence were found to be atypical form the global seasonality. Human RV-A infections started off with steadily increasing numbers of cases in the summer season and peaked at the fall season (around months of October/November). The number of positive cases then tapered off toward winter and after the January, it continued to decrease in the spring months to as low as 24 cases in the month of June (Figure 1).

### Age distribution

Human RV-A infections occurred mostly in children below the age of 5 years and the highest incidence rate (69.4%) was observed in infants below the age of one year. More than 96.8% of the patients were under two years old and above 50% aged 6 months or less. The mean of the infection incidence decreased continuously regarding the patients from 3 to 5 years old.

### VP4 and VP7 genotyping and molecular epidemiology

The VP4 genotyping showed that most 146/151 (96.7%) of positive samples were of genotype P [8], followed by genotypes P [4] 4/151 (2.6%), and P [6] 1 (0.66%), however, some samples n = 20/171 (11.7%) were untypeable after 2 or more attempts of amplification. The VP7 genotyping showed the following genotypes: G1 79/128 (61.7%), G2 7/128 (5.5%), G3 5/128 (3.9%), G4 5/128 (3.9%), G9 24/128 (18.8%), G12 8/128 (6.3%) and untypeable G were 43 samples out of 171 (25.1%). Overall, human RV-A genotyping and molecular epidemiology results showed that our regional RV-A combined G and P [types] were: G1P [8] 70/113 (61.9%); G9P [8] 19/113 (16.8%); G12P [8] 7/113 (6.2%); G3P [8] 5/113 (4.4%); G4P [8] 4/113 (3.5); G2P [8] 3/113 (2.6%); G2P [4] 3/113 (2.6%); G1P [4] 1/113 (0.88%); and G12P [6] 1/113 (0.88%) (Table 2). Hence, we identified the infrequent G1P [4] and G12P [6] genotypes were observed in 2/113 1.8% of infected Saudi children as a newly emerged genotype (Table 2).

**Table 2:** **Molecular combinations of G and [P] types of Rotavirus strains (total N = 113) detected between 2011 and 2012 among Saudi children with acute diarrhea admitted to KAMC-R**

**Genotype**	**N** ^**•**^	**%**
G1P [8]	70	61.9
G9P [8]	19	16.8
G12P [8]	7	6.2
G3P [8]	5	4.4
G4P [8]	4	3.5
G2P [8]	3	2.7
G2P [4]	3	2.7
G1P [4]	1	0.88
G12P [6]	1	0.88

^**•**^Total number of isolates were genotyped for both G and P was N = 113.

### Phylogenetic analysis

**Figure 2:**
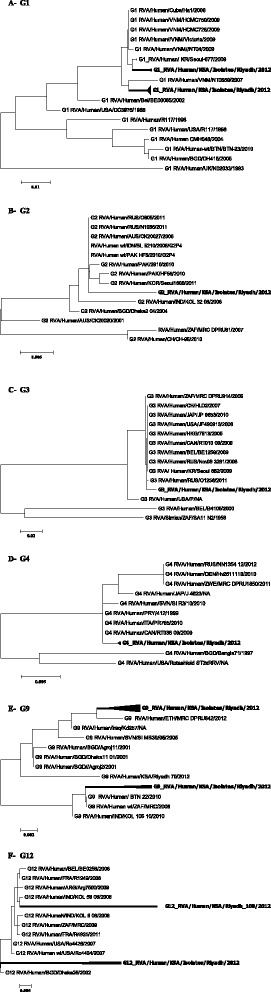
Phylogenetic trees of VP7 gene. (**A**-**F**) Trees were built using MEGA6 software with the maximum likelihood method and 1000 permutation for bootstrap. The scale bars indicating the nucleotide substitutions per site and the Saudi genotypes were highlighted in bold: A- G1; B- G2; C- G3, d- G4; E- G9 and F- G12.

**Figure 3:**
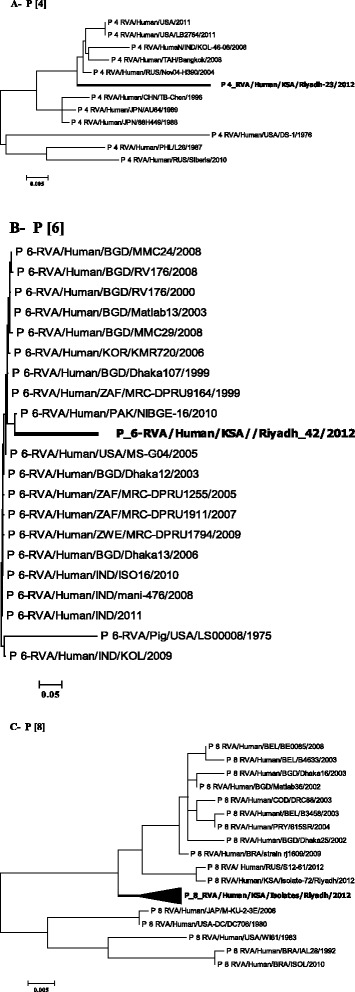
Phylogenetic trees of VP4 gene. (**A**-**C**) Trees were built using MEGA6 software with the maximum likelihood method and 1000 permutation for bootstrap. The scale bars indicating the nucleotide substitutions per site and the Saudi genotypes were highlighted in bold: A- P [4]; B- P [6] and C- P [8].

Phylogenetic trees based on the VP4 and VP7 sequence analysis revealed that G1, G2, G3, G4, G9 G12, P [4] and P [6] from Riyadh, Saudi Arabia were clustered within their related homologues strains from different countries such as: USA, Japan, China, India, Pakistan and Bangladesh with the following GenBank accession numbers (n = 80) AB118024; AB247943; AB585928; AB741657; AB905463; AF501578; AF528204; AY740736; AY787650; AY787652; D86271; DQ005112; DQ146646; DQ146652; DQ146654; DQ146667; DQ146679; DQ146684; DQ146697; DQ146699; DQ396443; DQ490539; DQ490546; DQ490551; DQ492669; DQ870505; DQ904524; EF554083; EF583046; EF672578; EF672584; EF672591; EF694178; EF694179; EF694184; FJ598026; GQ117006; GQ996883; GU985260; HF952915; HM035518; HM130969; JF490913; JF490979; JN088466; JN232048; JN258874; JN711097; JN711098; JN714986; JN827253; JN849118; JN849146; JQ069522; JQ069530; JQ087450; JQ837882; JX076836; JX195079; JX273720; JX841121; JX841124; KC443350; KC443768; KC580259; KC689824; KF414569; KF414603; KF614667; KF812586; KJ432717; KJ432745; KJ752344; KJ752410; KJ753186; KJ753373; KJ753484; KM008662; KP013536; U08431 (Figures 2 and 3).”

## Discussion

During the specified study period, we were able to identify certain newly emerging rotavirus genotypes introduced to Saudi Arabia, such as the uncommon G1P [4] and G12P [6] genotypes. Notably, there are at least 37 P types (P [1]-P [37]) and 27 G types (G1-G27) [10,11]. The common human RV-A genotypes that are circulating worldwide include: G1P [8], G2P [4], G3P [8], G4P [8] and G9P [8]. Globally, more than 90% of human RV-A cases have been attributed to G1P [8], G2P [4], G3P [8], and G4P [8] rotavirus genotypes. In Saudi Arabia, overall, rotavirus infection has a high prevalence (average: 33.7%) [18]. Globally and particularly in the northern hemisphere countries, human RV-A infections occur in clusters in the winter months. In the Middle East and the gulf region several countries reported rotavirus seasonal peak between November and April including Oman, Iran, Tunisia, Morocco and Turkey [19]. While others, Egypt and United Arab Emirates, recorded seasonal peak during July as well [20]. In our country, however, the season of human RV-A infections starts with steadily increasing numbers of cases in July and the incidence of the disease spikes up during chilly winter months. Rotavirus infection cases seem to have increased steadily in the region and the highest numbers were observed in 2012 as compared to the previous years (Balkhy et al. Manuscript in preparation). This consistent increase in the numbers of human RV-A infections can possibly be attributed to the absence of national RV vaccination program in this country. Children less than 2 years of age were the most vulnerable to human RV-A infections. Previously, Kheyami et al. showed that 89% of rotavirus strains were of G1P8 genotype [7,21-23]. Here, we found a consistent increase in the incidence of human RV-A infections during 2012 (30.6%) and the most prevalent genotypes were G1P [8] (61.5%) and G9P [8] (16.8%). We speculate that the emergence of the uncommon genotypes (≈5%), such as G1P [4] and G12P [6], could be due to either the expression of the natural variation of distinct human RV-A genotypes over time in rotavirus-infected Saudi children or they were circulating without detection. The phylogenetic analysis showed that G1P [4] and G12P [6], isolates from Riyadh, Saudi Arabia clustered with isolates from other countries such as Bangladesh, China, Japan, Thailand and Philippines. Nevertheless, it is possible that these genotypes were merging with the immigration of the foreign workers coming from Southeast Asia as the phylogenetic analysis has revealed. The divergent incidence of human RV-A infection and the fluctuating seasonal distribution of its genotypes underscore the need for a national RV vaccination program. As though the available vaccines do not cover all uncommon genotypes, however, their efficiency against various circulating human RV-A genotypes has been demonstrated in several studies [24-26]. Of note, rotavirus vaccines were recommended worldwide by the WHO in 2008; however, these have not been a part of routine Saudi vaccination program until 2013.

## Conclusions

Overall, the data show that 80% of our regional human RV-A genotypes included G1P [8] and G9P [8] whereas G1P [8] is the most prevalent (62%) rotavirus genotype in the region. In our region, the uncommon G1P [4] and G12P [6] genotypes were identified as the newly emerging RV strains. Further studies will be required to continuously monitor the seasonal prevalence and emergence of new RV genotypes in this country. In this regard, more advanced surveillance and in-depth analysis would be required, especially after the implementation of rotavirus vaccination in Saudi children. Close monitoring and regular reporting of the newly emerging genotypes would lie at the heart of a successful human RV-A infections/gastroenteritis control.
